# Fetal growth restriction: associated genetic etiology and pregnancy outcomes in a tertiary referral center

**DOI:** 10.1186/s12967-022-03373-z

**Published:** 2022-04-09

**Authors:** Meiying Cai, Na Lin, Linjuan Su, Xiaoqing Wu, Xiaorui Xie, Shiyi Xu, Xianguo Fu, Liangpu Xu, Hailong Huang

**Affiliations:** 1 Medical Genetic Diagnosis and Therapy Center, Fujian Maternity and Child Health Hospital, College of Clinical Medicine for Obstetrics & Gynecology and Pediatrics, Fujian Medical University, Fujian Key Laboratory for Prenatal Diagnosis and Birth Defect, Fuzhou, China; 2 Guangxi Medical University, Guangxi, China; 3 Department of Prenatal Diagnosis, Ningde Municipal Hospital, Ningde Normal University, Ningde, China

**Keywords:** Fetal growth restriction, SNP-array, Karyotype analysis, Copy number variation

## Abstract

**Background:**

The etiology of fetal growth restriction (FGR) is complex and currently, there is a paucity of research about the genetic etiology of fetal growth restriction. We investigated the genetic associations and pregnancy outcomes in cases of fetal growth restriction.

**Methods:**

A retrospective analysis of 210 pregnant women with fetal growth restriction was performed using karyotype analysis and single nucleotide polymorphism arrays (SNP-array). The differences in pathogenic copy number variation (CNV) detected by the two methods were compared. At the same time, the fetuses were divided into three groups: isolated FGR (n = 117), FGR with ultrasonographic soft markers (n = 48), and FGR with ultrasonographic structural anomalies (n = 45). Further, the differences in pathogenic copy number variations were compared among the groups.

**Results:**

The total detection rate of pathogenic CNVs was 12.4% (26/210). Pathogenic copy number variation was detected in 14 cases (6.7%, 14/210) by karyotype analysis. Furthermore, 25 cases (11.9%, 25/210) with pathogenic CNVs were detected using the SNP-array evaluation method. The difference in the pathogenic CNV detection rate between the two methods was statistically significant. The result of the karyotype analysis and SNP-array evaluation was inconsistent for 13 cases with pathogenic CNV. The rate of detecting pathogenic CNVs in fetuses with isolated FGR, FGR combined with ultrasonographic soft markers, and FGR combined with ultrasonographic structural malformations was 6.0, 10.4, and 31.1%, respectively, with significant differences among the groups. During the follow-up, 35 pregnancies were terminated, two abortions occurred, and 13 cases were lost to follow-up. Of the 160 deliveries, nine fetuses had adverse pregnancy outcomes, and the remaining 151 had normal postnatal growth and developmental assessments.

**Conclusions:**

Early diagnosis and timely genomic testing for fetal growth restriction can aid in its perinatal prognosis and subsequent intervention.

**Supplementary Information:**

The online version contains supplementary material available at 10.1186/s12967-022-03373-z.

## Background

Fetal growth restriction (FGR) refers to the condition of inadequate growth of a fetus due to a variety of factors. The American College of Obstetricians and Gynecologists defines FGR in terms of fetal birth mass that is below the 10th percentile of the average body mass for a child of the same gestational age [1]. FGR is a common obstetric complication that is associated with premature delivery, fetal death in utero, neonatal death, and other adverse outcomes [2, 3]. Hence, preventing FGR is significant in improving the pediatric outcomes. However, the etiology of FGR is complex, and can be caused by maternal, fetal, placental, and umbilical cord factors [3]. These factors do not allow the fetus to receive adequate energy and nutrients for growth and development [4].

It is essential to identify the etiology of FGR for better diagnosis and providing possible treatments for this condition. Previous research focused on diagnosis, intrauterine monitoring, treatment, and prognosis of fetuses with FGR; however, currently there are only a few studies on the genetic etiology of FGR. Genetic factors that cause FGR have rarely been reported, and some studies that reported a genetic association were conducted with small sample sizes [5]. Single-nucleotide polymorphism array (SNP-array) can detect copy number variations (CNVs) at a genome-wide level, as well as chimeras (> 30%), loss of heterozygosity, and uniparental disomy (UPD) [6, 7]. SNP-array has been widely used in the diagnosis of fetal structural malformations, primary mental retardation, growth and developmental retardation, autism, and tumors [8, 9]. In this study, we utilized karyotyping and SNP-array for the genomic analysis of 210 fetuses who were prenatally diagnosed with FGR using ultrasound, and investigate the genetic etiology of FGR and evaluate the diagnostic value of SNP-array. The outcomes of these pregnancies were also monitored.

## Methods

### Patient data

This study retrospectively enrolled 210 pregnant women, who received a prenatal diagnosis of FGR by fetal ultrasound at a tertiary care center between November 2016 and February 2021. The gestational age range was 16–35+6 weeks and the maternal age range was 17–48 years. Depending on the gestational age, amniotic fluid or cord blood samples were collected for karyotype analysis and SNP-array evaluation. The inclusion criteria were as follows: Gestational age determined based on the date of last menstruation, a detailed menstrual history, and ultrasound examination during the first trimester. Fetal abdominal circumference, head circumference, biparietal diameter, and femur length were measured by ultrasound, and the estimated fetal weight was calculated using the Hadlock formula. FGR was defined as a fetal mass below the 10th percentile of the average weight of a normal fetus of the same gestational age [10]. The exclusion criteria were as follows: FGR resulting from a clear maternal cause, such as hypertensive disease during pregnancy, maternal infection, or maternal nutritional abnormalities.

**Fig.1:**
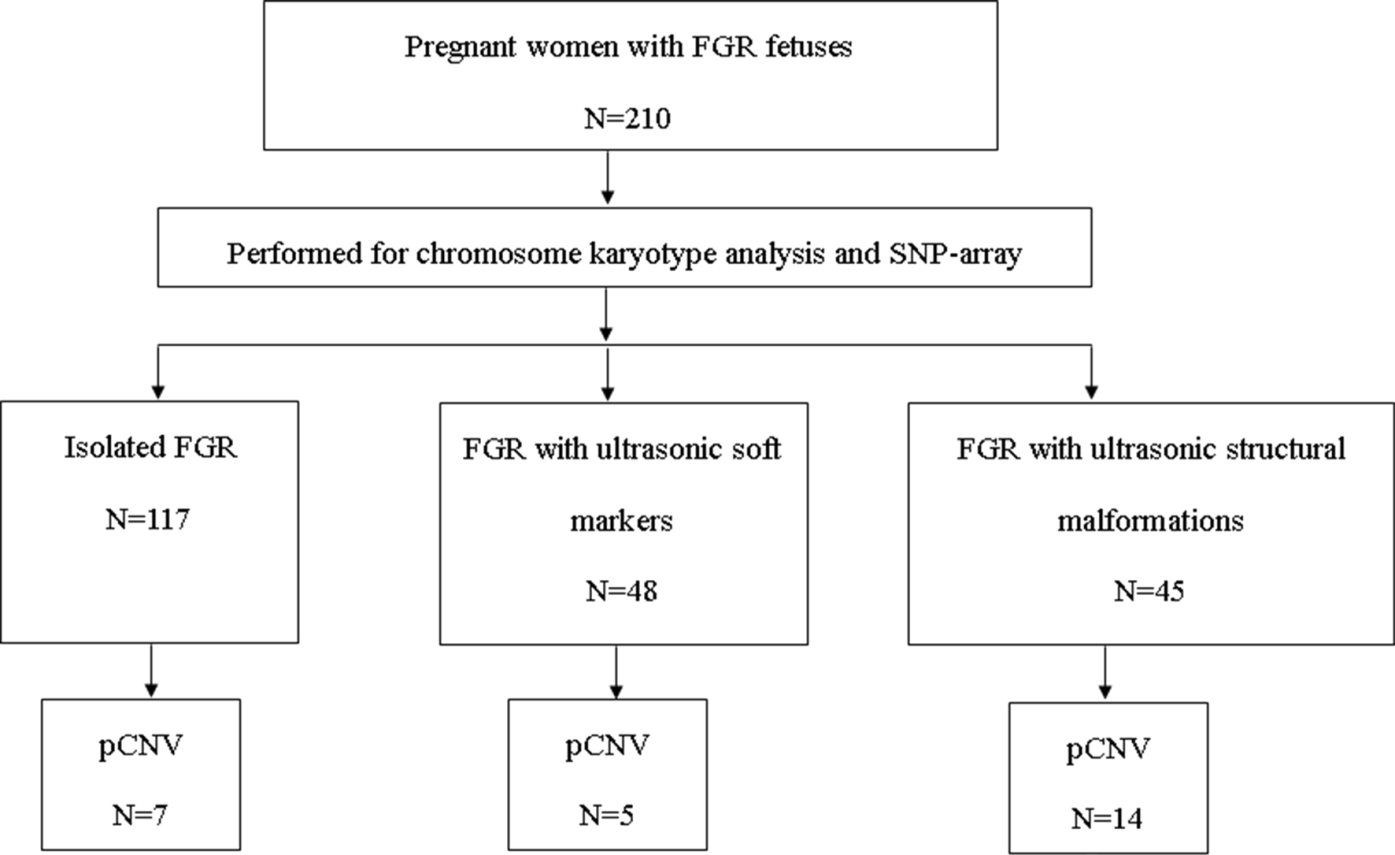
Enrollment of Study Participants between November 2016 and February 2021

Depending on the co-occurrence of FGR with other ultrasonographic abnormalities into, the patients in this study were divided into three groups i.e., isolated FGR group (117 cases), FGR with ultrasonographic soft markers (48 cases) group, and FGR with ultrasonographic structural malformations (45 cases) group (Fig. 1). Ultrasonographic soft markers refer to abnormalities other than the ultrasonic structure found in the fetal ultrasound examination. They are non-specific indicators and cannot completely indicate fetal structural abnormalities, and may just be normal variations.

### Karyotype analysis

Using ultrasonography, 30 mL amniotic fluid was extracted. Of this, 20 mL of the fluid was used for cell culture and 10 mL was used for SNP-array. Alternatively, 2.5 mL of cord blood was extracted under ultrasonographic guidance, of which 1.5 mL was used for cell culture and 1.0 mL was used for SNP-array. We also collected 2 mL of peripheral blood from all the pregnant women and their spouses in EDTA anticoagulant tubes. Amniotic fluid or umbilical cord blood samples were extracted and inoculated in RPMI-1640 culture medium (Ningbo Bosheng Medical Technology). Cells were harvested from umbilical cord blood samples after 3 days of culture, and from amniotic fluid samples after 8 days of culture. Thereafter, samples were mounted on drop slides and Giemsa staining was conducted (if necessary, C band and N band were also included). Finally, karyotypes were identified using the GSL-120 automatic chromosome scanning platform (Leica Biosystems, Wetzler, Germany), and karyotype calculation and analysis was performed. Karyotypes were named according to the International System for (Human) Cytogenetic Nomenclature 2016. Forty karyotypes were counted in each case, five were analyzed, and 20 more karyotypes were counted and analyzed in case of any abnormality.

### Single-nucleotide polymorphism array

Affymetrix® (Affymetrix, Santa Clara, CA, USA) microarray chips and CytoScan™ (Thermo Fisher Scientific Inc., Waltham, MA, USA) 750 K microarray reagent were used for the SNP-array. Digestion, ligation, amplification, purification, labeling, hybridization, washing, and scanning of DNA samples was conducted according to the standard operating procedures provided by the company. Affymetrix Genechip Microarray Instrumentation System (Affymetrix, California, USA) and the supporting Chromosome Analysis Suite software were used to analyze the data. The SNP results were further analyzed using related databases. The databases used included the international public DGV benign variation database (http:// projects. The tcag. Ca/variation), the international public DECIPHER pathological variation database (https://www.deciphergenomics.org/), the online Human Mendelian genetic database OMIM (http://www.omim.org), the international federation of cell gene chip standardized ISCA (https://www.iscaconsortium.org/), and the global Affymetrix pathological shared database user CAGdb (http://www.cagdb.org/), NCBI, PubMed, etc. The nature of CNVs was determined according to standards and guidelines of the American Society of Medical Genetics for the interpretation and reporting of genetic CNVs. CNVs can be roughly divided into five categories: pathogenic CNV, likely pathogenic CNV, variants of uncertain clinical significance (VUS) CNV, likely benign CNV, and benign CNV [11, 12]. For VUS CNV, it recommended to perform an SNP-array using the parents’ blood samples, combined with a pedigree analysis to further clarify the nature of the CNV.

### Pregnancy outcome

All cases were followed up telephonically to obtain information regarding fetal development, pregnancy outcome, and postpartum growth and development.

### Statistical analysis

IBM SPSS Statistics for Windows, version 25 (IBM Corp., Armonk, N.Y., USA) was used for statistical analyses. Chi-square test was used for comparison between groups for categorical variables. Statistical significance was set at p < 0.05.

## Results

### Karyotype analysis

Among 210 cases of FGR, 14 (6.7%, 14/210) had abnormal karyotypes, including nine cases with abnormal chromosomal numbers and five with abnormal structure (Table 1). The parents of fetuses with abnormal karyotypes chose to terminate the pregnancy following genetic counseling. Among nine cases, there were two cases of trisomy 21, two of trisomy 18, three of a sex chromosome abnormality, and two cases of chimeric abnormality. The five cases of chromosomal structural abnormalities consisted of one case of partial deletion, three cases of partial duplication, and one case of chimeric structural abnormalities. Among the 14 abnormal karyotypes, six cases were of isolated FGR and eight cases were of FGR with miscellaneous ultrasonographic abnormalities. The most common ultrasonographic abnormality with FGR was congenital heart disease (five cases). The other three cases had thickened nuchal translucency, small size of the kidneys, and facial deformity, respectively.

**Table 1:** Abnormal karyotyp analysis results of fetuses with FGR

Case	Karyotype	SNP-array results	Prenatal ultrasound	Postnatal outcome
1	47,XXX	arr[hg19] (X) × 3	FGR	TP
2	47,XXX	arr[hg19] (X) × 3	FGR	TP
3	45,X	arr[hg19] (X) × 1	FGR	TP
4	47,XX, + 9[15]/46,XX[8]	arr[hg19] (9) × 2 ~ 3	FGR	TP
5	47,XXX[62]/45,X[18]	Normal	FGR	TP
6	47,XX,psvidis(9)(q12)[39]/46,XX[11]	arr[hg19] 9p24.3q13(208,454–68,216,577) × 4	FGR	TP
7	46,XX,dup(12)(q14q23)	arr[hg19] 12q14.2q23.1(64,877,459–97,710,202) × 3	FGR, thickened nuchal translucency	TP
8	47,XY, + 21	arr[hg19](21) × 3	FGR, VSD	TP
9	47,XX, + 21	arr[hg19](21) × 3	FGR, VSD, renal parenchyma echo enhancement	TP
10	47,XX, + 18	arr[hg19](18) × 3	FGR, VSD	TP
11	47,XX, + 18	arr[hg19](18) × 3	FGR, VSD, nasal bones small, overlapping fingers	TP
12	46,XY,add(16)(p13.3)	arr[hg19]16p13.3(85,880–536,631) × 3	FGR, VSD	TP
13	46,XY,del(4)(p15)	arr[hg19] 4p16.3p15.1(68,345–35,252,743) × 1	FGR, small kidneys	TP
14	46,XX,add(12)(q24)	arr[hg19]11q23.2q25(113,998,447–134,937,416) × 3, 12q24.33(133,718,370–133,777,562) × 1	FGR, small mandible, mandible retraction	TP

Among the nine cases, there were two cases of trisomy 21, two of trisomy 18, three of sex chromosome abnormality, and two cases of chimeric abnormality. *VSD* ventricular septal defect, *SNP* single-nucleotide polymorphism, *FGR* fetal growth restriction, *TP* termination of pregnancy

### Single-nucleotide polymorphism array

Of the 210 samples, SNP-array analysis detected 32 abnormal cases, including 25 cases of pathogenic CNV (11.9%, 25/210), five cases of VUS CNV (2.4%, 5/210), and two cases of benign CNV (1.0%, 2/210) (Tables 1 and 2). The 25 cases of pathogenic CNV included eight cases of aneuploidy, 14 cases of partial deletion/duplication, one case of chimerism, and three cases of UPD.

**Table 2:** SNP-array results of fetal FGR with normal karyotype analysis

Case	SNP array locus	Size (Mb)	Prenatal ultrasound	Pathogenicity classification	Postnatal outcome	Inheritance
1	arr[hg19]7q11.23(72,723,370–74,143,240) × 1	1.4	FGR	P	TP	–
2	arr[hg19]15q14q21.3(35,077,111–54,347,324)hmz	19.2	FGR, polyhydramnios	P (Prader-Willi)	TP	UPD (Maternal)
3	arr[hg19]10q11.22q11.23(46,252,072–51,903,756) × 1	5.6	FGR, echogenic bowel	P	TP	de novo
4	arr[hg19]8q11.23q12.1(54,456,444–59,599,862) × 1	5.1	FGR, single umbilical artery, oligohydramnios	Likely P	TP	–
5	arr[hg19]6p25.3q27(203,877–170,896,644) × 2 hmz	–	FGR, echogenic bowel, mild tricuspid regurgitation, reverse ductus alpha wave	P	TP	UPD (Paternal)
6	arr[hg19]4p16.3p16.1(68,345–6,608,624) × 1	6.5	FGR, pulmonary stenosis	P (Wolf-Hirschhorn)	TP	–
7	arr[hg19]15q24.1q24.2(72,965,465–75,567,135) × 1	2.6	FGR, VSD, pulmonary valve stenosis with insufficiency	P (15q24 microdeletion syndrome)	TP	de novo
8	arr[hg19]16q23.3q24.3(79,800,878–90,146,366) × hmz,16p13.3p12.3(94,807–19,302,326) × hmz	10.3 19.2	FGR, VSD, aortarctia, left kidney dysplasia	Likely P	TP	–
9	arr[hg19]22q11.21(18,648,855–21,459,713) × 3	2.8	FGR, intrahepatic portal shunt, umbilical vein tumor like dilatation	P (22q11.21 microduplication)	TP	Maternal
10	arr[hg19]22q11.21(18,648,855–21,800,471) × 1	3.1	FGR, VSD	P (22q11.21 microdeletion syndrome)	TP	–
11	arr[hg19]17p11.2(16,615,982–18,922,171) × 3	2.1	FGR, VSD	P (Potocki-Lupski)	TP	Maternal
12	arr[hg19]2p25.3p11.2(50,813–87,053,152)hmz,arr[hg19]2q11.1q37.3(95,550,957–242,773,583) hmz	–	FGR, persistent left superior vena cava, renal parenchyma echo enhancement	Likely P	TP	UPD (Maternal)
13	arr[hg19]22q11.21(18,648,855–21,800,471) × 3	3.1	FGR	VUS	TD	Paternal
14	arr[hg19]4q28.3q31.3(133,718,289–154,569,367)hmz	20.8	FGR	VUS	TD	–
15	arr[hg19]15q24.1q24.2(72,969,435–75,601,120) × 3	2.6	FGR	VUS	TD	Maternal
16	arr[hg19]14q12(25,364,014–26,860,808) × 3	1.4	FGR	VUS	TD	–
17	arr[hg19]4q24(106,284,925–107,545,257) × 3	1.2	FGR, VSD	VUS	–	–
18	arr[hg19]18p11.23p11.22(7,153,845–8,964,650) × 3	1.8	FGR	Likely B	TD	Paternal
19	arr[hg19]4q35.2(188,155,530–190,957,460) × 1	2.8	FGR, tricuspid regurgitation	Likely B	TD	Maternal

The 19 cases with normal karyotype analysis but SNP-array abnormality included 12 pathogenic CNV, 5 VUS and 1 benign CNV. *VSD* ventricular septal defect, *SNP* single-nucleotide polymorphism, *FGR* fetal growth restriction, *P* pathogenic, *B* benign, *TP* termination of pregnancy, *TD* term delivery

Of the 32 abnormal cases detected by the SNP-array, 19 cases were normal as per karyotype analysis (Table 2), including six cases of isolated FGR and 13 cases of FGR with miscellaneous ultrasonographic abnormalities. Among the 13 cases with miscellaneous ultrasonographic abnormalities, there were six cases of ventricular septal defect. The 19 cases with normal karyotype analysis but abnormal SNP-array, included 12 cases of pathogenic CNVs, five VUS CNVs, and one benign CNV. The parents of 12 fetuses with pathogenic CNV chose to terminate the pregnancy following genetic counseling. The parents of five fetuses with VUS CNV and one fetus with benign CNV chose to continue the pregnancy.

### Comparison of karyotype analysis and SNP-array results

**Fig.2:**
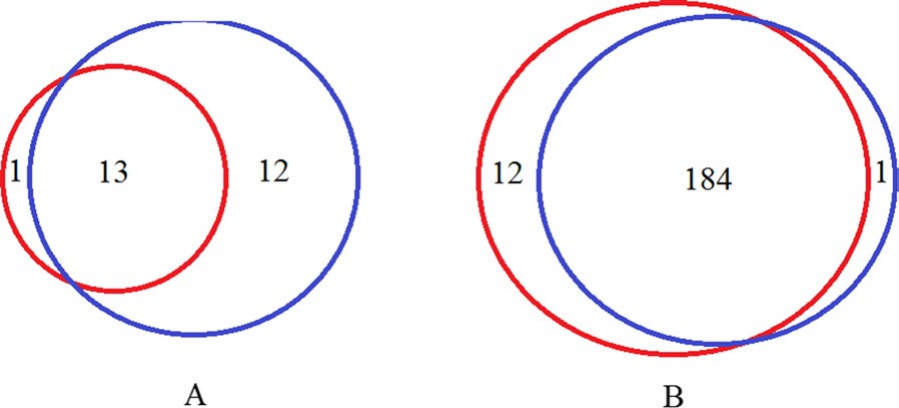
The consistent and inconsistent cases for karyotype analysis and SNP-array. The blue circle represents SNP-array analysis. The red circle represents karyotype analysis. A shows abnormal results detected by karyotype analysis and SNP-array. B shows normal results detected by karyotype analysis and SNP-array

The total detection rate of pathogenic CNVs was 12.4% (26/210) using karyotype analysis and SNP-array. The detection rates of pathogenic CNVs by karyotype analysis alone, and SNP-array alone, were 6.7% (14/210) and 11.9% (25/210), respectively, and the difference between the two methods was statistically significant (χ^2^ = 3.42, P = 0.046). The results of karyotype analysis and SNP-array were inconsistent in 13 of the 210 samples (Fig. 2), including one case of a low proportion of sex chromosome chimera, nine cases of chromosome microduplication or microdeletion, and three cases of UPD. The low proportion of sex chromosome chimera was detected as normal by the SNP-array analysis. Microduplication, microdeletion, and UPD were normal upon karyotype analysis.

### Comparison of pathogenic CNVs in different FGR groups

**Fig. 3:**
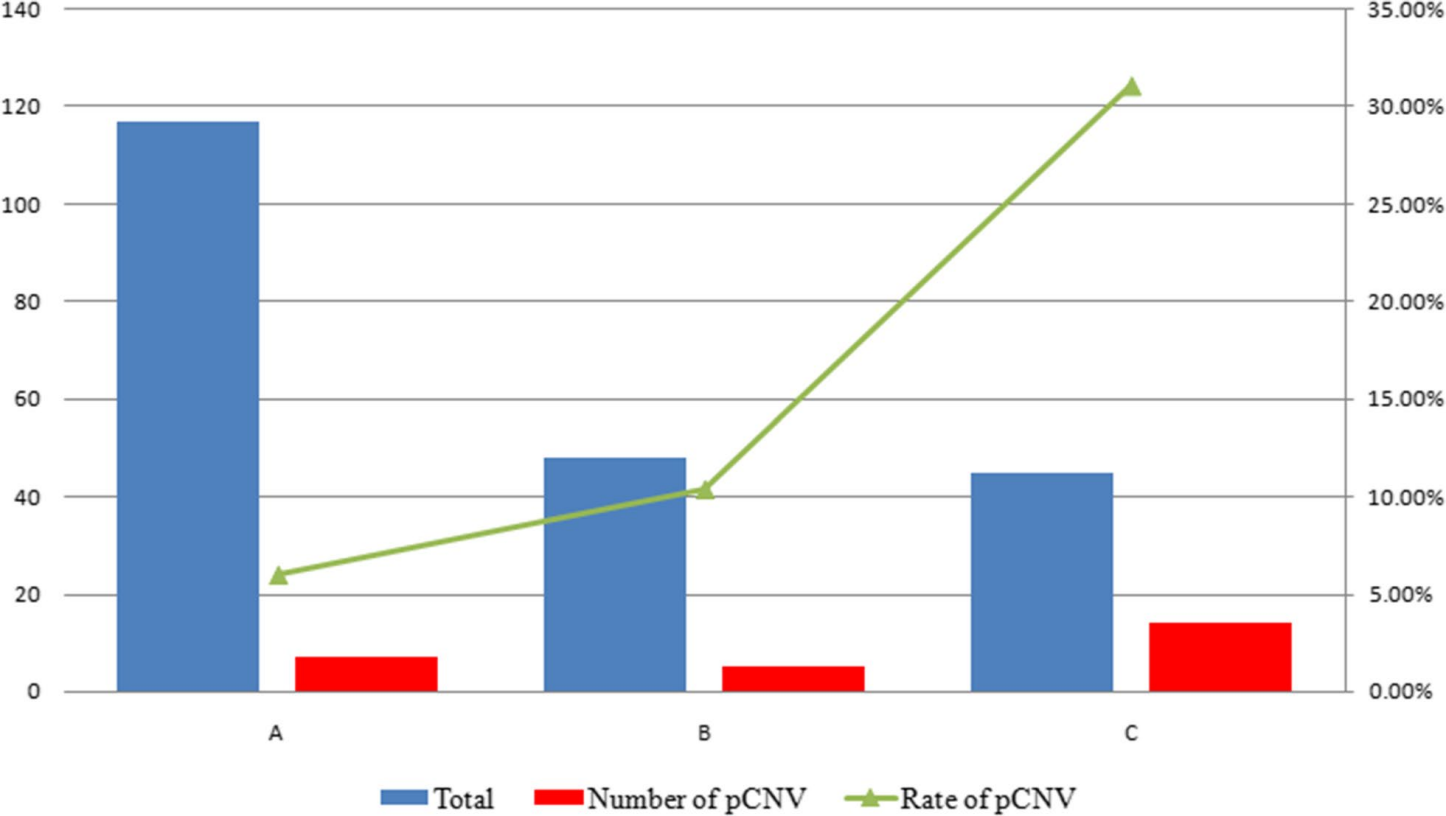
Comparison of pathogenic CNVs in fetuses with FGR among three groups. **A** isolated FGR group; **B** FGR with ultrasonographic soft markers group; **C** FGR with ultrasonographic structural anomalies. The difference in pathogenic CNVs among the three groups was statistically significant

Fetuses with FGR were divided into three groups according to the presence of ultrasonographic abnormalities: isolated FGR group, FGR with ultrasonographic soft markers group, and FGR with ultrasonographic structural anomalies group. For the group with isolated FGR (n = 117), seven cases of pathogenic CNV were detected with a positivity rate of 6.0%. For FGR with ultrasonic soft markers (n = 48), five cases of pathogenic CNV were detected with a positivity rate of 10.4%. There were 45 cases of FGR with ultrasonographic structural anomalies, and 14 cases of pathogenic CNVs were detected with a positivity rate of 31.1%. The difference in pathogenic CNVs among the three groups was statistically significant (χ^2^ = 19.138, P = 0.00007) (Fig. 3).

### Follow-up and pregnancy outcomes

The 210 FGR cases were followed up to assess the outcomes and to document postnatal development (Table 3). Among them, 35 pregnancies were terminated (nine with normal karyotype analysis and SNP-array results, 26 with pathogenic CNVs), two fetuses were aborted (normal karyotype analysis and SNP-array results), and 13 were lost to follow-up. Of the 160 deliveries, four infants died within 5 months, four were dwarf after birth, one infant was diagnosed with a cardiac defect after birth, and 151 infants demonstrated normal growth and development after birth (Additional file 1).

**Table 3:** Pregnancy outcomes of 210 fetuses with FGR

SNP-array	Karyotype analysis	Pregnancy outcomes						Total (n)
		TP (n)	NP (n)	AP (n)	Aborted (n)	Postnatal deaths (n)	Loss follow-up (n)	
Normal	Normal	9	151	5	2	4	13	184
pCNV	Normal	12	0		0	0	0	12
Normal	Low proportion of SCA	1	0	0	0	0	0	1
pCNV	Aneuploid	8	0		0	0	0	8
pCNV	Structural abnormalities	5	0		0	0	0	5

Among 210 FGR cases, 35 pregnancies were terminated, 2 fetuses were aborted, and 13 were lost to follow-up. Of the 160 deliveries, four infants died after 5 months, four were dwarf after birth, one infant had a heart malformation after birth, and 151 infants had normal growth and development after birth. *AP* abnormal phenotype, *NP* normal phenotype, *TP* termination of pregnancy

## Discussion

Karyotype analysis can detect both numerical and structural chromosomal anomalies. In this study, karyotype analysis detected eight cases of abnormal chromosome number and six cases of structural chromosomal abnormality in FGR fetuses. Conventional karyotype analysis can only detect abnormal results in chromosomes larger than 10 Mb, but cannot detect minor structural chromosomal anomalies. SNP-array analysis can detect microdeletions, microduplications, and UPD, but cannot detect balanced translocation chromosomal abnormalities and low proportion chimerism [13]. In this study, SNP-array analysis was used to identify not only pathogenic CNVs that were detected by karyotype analysis, but also nine additional microdeletions or microduplications of less than 5 Mb in size as well as three cases of UPD. The detection rates of pathogenic CNVs in cases of FGR detected using karyotype analysis and SNP-array evaluation were 6.7% (14/210) and 11.9% (25/210), respectively, and the results were statistically significant. Therefore, SNP-array may have advantages for etiological investigation of FGR [14, 15]. However, it should be noted that the karyotype analysis result of a specific FGR case was low proportion of sex chromosome chimera, while the SNP-array for the same case was normal. Karyotype analysis may have an advantage over SNP-array analysis in detecting low proportions of chimera. Therefore, a combination of karyotype analysis and SNP-array analysis is recommended for the evaluation of genetic etiologies in cases of FGR.

The pathogenesis and mechanisms involved in FGR are complex. It has been suggested that approximately 7% of FGR is caused as a result of chromosome aneuploidy [15–17]. In this study, the chromosome aneuploidy rate in cases of FGR was 4.3% (9/210), which was slightly lower than that reported in previous studies. It has been reported that the genetic etiology of FGR is not only related to structural chromosomal abnormalities or aneuploidy, but also to chromosomal microdeletion/microduplication [2, 18]. Gruchy et al. performed chromosome microarray analysis (CMA) on 38 pregnant women with FGR or/combined multiple fetal malformations and found that CMA could detect 8% of meaningful chromosomal abnormalities under normal karyotype analysis [19]. Using SNP-array evaluation, we detected an additional 5.7% (12/210) pathogenic genomic abnormalities, which was slightly less than the value reported earlier. Chen et al. [20] proposed that 22q11.2 microdeletion syndrome in a fetus can likely lead to FGR combined with congenital heart disease. In our study, 22q11.2 microdeletion syndrome was detected in one FGR fetus with congenital heart disease, while 22q11.2 microduplication syndrome was detected in another FGR fetus along with other structural malformations. Similarly, Wolf-Hirschhorn syndrome is a common genomic disease that can reportedly cause FGR [18, 21]. The main locus contributing to FGR is on chromosome 4p16.3, and *TACC3* [22] and *SLBP* [23], which are found in the very same region, may be candidate genes for FGR. In our study, an FGR fetus with CNV microdeletion (6.5 MB) on chromosome 4p16.3 was identified, and ultrasonography detected FGR and pulmonary stenosis in the fetus.

Changes in the gene imprinting regions of some chromosomes and UPD may also lead to FGR. Three cases in this study had UPD. Further, chromosome 6q24 has gene imprinting regions [24], including *PLAG1* and *HYMAI* genes, and abnormalities in these may lead to fetal intrauterine growth retardation, temporary neonatal diabetes, macroglossia, or umbilical hernia. SNP-array analysis confirmed the existence of heterozygous deletion on chromosome 6 in a fetus, and paternal UPD was identified via pedigree analysis. Fetal ultrasonography in this case revealed FGR, echogenic bowel, mild tricuspid regurgitation, and reverse ductus alpha wave.

Prader-Willi syndrome (PWS) is a typical example of imprinting inheritance [25]. Maternal UPD on chromosome 15 can lead to PWS, and the main intrauterine manifestations of PWS are reduced fetal movement and FGR [26]. This study confirmed that one fetus had maternal UPD on chromosome 15 (PWS), and ultrasonographic findings of this fetus revealed FGR and polyhydramnios. Moreover, imprinted genes on chromosome 2 [27] are also associated with fetal growth restriction, growth retardation, heart malformation, hypospadias, and oligohydramnios. In our study, chromosome 2 of one of the fetuses exhibited maternal UPD, and ultrasonography demonstrated FGR, persistent left superior vena cava, and renal parenchyma echo enhancement in the fetus. These cases confirmed that the genetic etiology of FGR was not only related to chromosomal structural abnormalities or aneuploidy but also chromosomal submicroscopic abnormalities and UPD.

An et al. [28] carried out invasive prenatal diagnoses of isolated FGR fetuses and found that the detection rate of chromosomal karyotype abnormalities was 9.4%, among which the detection rate of CMA was 5.5%. Borrell et al. [29] conducted a meta-analysis using CMA analysis of FGR fetuses with normal karyotypes and found that that the abnormal detection rate of CMA in fetuses with isolated FGR and normal karyotype was 4%. In this study, fetuses were divided into isolated FGR group—FGR with ultrasonographic soft markers and FGR with ultrasonographic structural anomalies. The rates of pathogenic CNVs in fetuses with isolated FGR, FGR combined with ultrasonographic soft markers, and FGR combined with ultrasonographic structural anomalies were 6.0%, 10.4%, and 31.1%, respectively. FGR with ultrasonographic structural anomalies had a high rate of pathogenic CNVs due to high prevalence of fetal anomalies caused by genomic abnormalities. This study showed that the group with isolated FGR had 6% pathogenic CNVs. Therefore, identification of genetic causes in FGR using karyotype analysis and SNP-array analysis is recommended, irrespective of whether the FGR is isolated or combined with other ultrasonographic findings.

This study had certain limitations. Firstly, the samples were not analyzed based on age group, and maternal age may also be a factor influencing FGR. Secondly, the diagnosis of FGR was based on the ultrasonographic measurement standard at our institution, which may have some variation. Thirdly, the participants in this study were more likely to terminate their pregnancies after being informed of the presence of fetal pathogenic CNVs; thus, there was a lack of long-term follow up to evaluate the prognoses of the cases being investigated. VUS is challenging for laboratory technicians and clinical consultants—the detection rate of VUS is dependent on the type of microarray chip and the study population [30, 31]. According to literature, VUS accounts for 5.2% of FGR cases [30]. In this study, VUS accounted for 2.4% (5/210) of the FGR cases, which was lower than that reported previously. The five fetuses with VUS in this study developed well das determined during postnatal follow-ups, while nine FGR cases with normal karyotype and abnormal SNP-array had adverse pregnancy outcomes. Next-generation sequencing may provide a comprehensive prenatal diagnostic tool for use in cases of FGR as a new technique for the detection of single gene mutations and CNVs, and allow for improved evaluation of fetal prognosis.

## Conclusion

As a prenatal diagnostic tool in FGR, SNP-array analysis is not only conducive to the discovery of FGR-related genomic abnormalities but also for accurate assessment of fetal prognosis, which can aid in genetic counseling. Early diagnosis of FGR and timely detection of genomic abnormalities will positively impact fetal intervention and perinatal prognosis in cases of FGR. Such diagnostic modalities can make it possible to prevent the etiology of FGR and reduce its consequences.

## Supplementary Information

**Additional file 1.** Detailed supplemental material to cases for abnormal karyotype analysis results of fetuses with FGR.

## Data Availability

All data generated during and/or analyzed during the current study are available upon request by contact the corresponding author**.**

## Abbreviations

FGR: Fetal growth restriction
SNP-array: Single-nucleotide polymorphism array
CNVs: Copy number variations
CNVs: Copy number variations
