# Supplementary material for: Fetal growth restriction: associated genetic etiology and pregnancy outcomes in a tertiary referral center
Source: J Transl Med. 2022 Apr 9;20:168. doi: 10.1186/s12967-022-03373-z (PMC8994287; doi:10.1186/s12967-022-03373-z)
Supplement: Supplementary file 1 — Additional file 1. Detailed supplemental material to cases for abnormal karyotype analysis results of fetuses with FGR. [file 12967_2022_3373_MOESM1_ESM.docx]

Detailed supplemental material to cases for abnormal karyotype analysis results of fetuses with FGR

Case 1: Prenatal ultrasound phenotype was isolated FGR. Karyotype analysis shows 47,XXX. SNP-array shows arr[hg19] (X)x3. This aneuploidy is a definite pathogenic CNVs. After genetic counseling, the parents of the fetus chose to terminate the pregnancy.

Case 2: Prenatal ultrasound phenotype was isolated FGR. Karyotype analysis shows 47,XXX. SNP-array shows arr[hg19] (X)x3. This aneuploidy is a definite pathogenic CNVs. After genetic counseling, the parents of the fetus chose to terminate the pregnancy.

Case 3: Prenatal ultrasound phenotype was isolated FGR. Karyotype analysis shows 45,X. SNP-array shows arr[hg19] (X)x1. This aneuploidy is a definite pathogenic CNVs. After genetic counseling, the parents of the fetus chose to terminate the pregnancy.

Case 4: Prenatal ultrasound phenotype was isolated FGR. Karyotype analysis shows 47,XX,+9[15]/46,XX[8]. SNP-array shows arr[hg19] (9)x2~3. This aneuploidy is a definite pathogenic CNVs. After genetic counseling, the parents of the fetus chose to terminate the pregnancy.

Case 5: Prenatal ultrasound phenotype was isolated FGR. Karyotype analysis shows 47,XXX[62]/45,X[18]. SNP-array shows normal. Low rates of chimeric sex chromosome abnormalities may have a pathogenic phenotype or a normal phenotype. After genetic counseling, the parents of the fetus chose to terminate the pregnancy.

Case 6: Prenatal ultrasound phenotype was isolated FGR. Karyotype analysis shows 47,XX,psvidis(9)(q12)[39]/46,XX[11]. SNP-array shows arr[hg19] 9p24.3q13(208,454-68,216,577)x4. Affymetrix CytoScan 750K Array showed that there was an increase in genomic copy number (4) in p24.3 q13 region of chromosome 9, involving a fragment size of about 68Mb, containing 149 OMIM genes. Literature and database query, confirm the area increased copy number can lead to 9 p tetraploid syndrome, and patients' main clinical manifestation is serious growth retardation, mental retardation, characteristic face, eyes wide, and low set ears, small jaw. It can also be combined cardiac malformations, congenital anomalies, such as cleft lip. Therefore, the genomic copy number variation in this case is a pathogenic variation. After genetic counseling, the parents of the fetus chose to terminate the pregnancy.

Case 7: Prenatal ultrasound phenotype was FGR and thickening of the NF. Karyotype analysis shows 46,XX,dup(12)(q14q23). SNP-array shows arr[hg19] 12q14.2q23.1(64,877,459-97,710,202)x3. Afymetrix CytoScan 750K Array showed that duplication of genomic copy number in q14.2q23.1 region of chromosome 12, involving a fragment size of about 32.8Mb, containing 107 OMIM genes. According to literature review and database, multiple case reports suggest that duplication of copy number in this region can cause congenital abnormalities, mainly manifested as developmental delay, intellectual disability, cognitive impairment, behavioral affective disorder, visual impairment and dystonia. The duplication in copy number on chromosome 12 was confirmed as a new mutation by pedigree verification. The clinical significance is pathogenic. After genetic counseling, the parents of the fetus chose to terminate the pregnancy.

Case 8: Prenatal ultrasound phenotype was FGR and VSD. Karyotype analysis shows 47,XY,+21. SNP-array shows arr[hg19](21)×3. This aneuploidy is a definite pathogenic CNVs. After genetic counseling, the parents of the fetus chose to terminate the pregnancy.

Case 9: Prenatal ultrasound phenotype was FGR, VSD , and renal parenchyma echo enhancement. Karyotype analysis shows 47,XY,+21. SNP-array shows arr[hg19](21)×3. This aneuploidy is a definite pathogenic CNVs. After genetic counseling, the parents of the fetus chose to terminate the pregnancy.

Case 10: Prenatal ultrasound phenotype was FGR and VSD. Karyotype analysis shows 47,XX,+18. SNP-array shows arr[hg19](18)×3. This aneuploidy is a definite pathogenic CNVs. After genetic counseling, the parents of the fetus chose to terminate the pregnancy.

Case 11: Prenatal ultrasound phenotype was FGR, VSD, nasal bones small, and overlapping fingers. Karyotype analysis shows 47,XX,+18. SNP-array shows arr[hg19](18)×3. This aneuploidy is a definite pathogenic CNVs. After genetic counseling, the parents of the fetus chose to terminate the pregnancy.

Case 12: Prenatal ultrasound phenotype was FGR and VSD. Karyotype analysis shows 46,XY,add(16)(p13.3). SNP-array shows arr[hg19]16p13.3(85,880-536,631)x3. Afymetrix CytoScan 750K Array showed that there was a gene copy number deletion in p13.3 region of chromosome 16, with a copy number of 1. The involved fragment size was about 451Kb, containing 17 OMIM genes. There are multiple cases in the database with physical retardation, mental retardation, characteristic facial features, seizures, microcephaly and other congenital abnormalities. The clinical significance is pathogenic. After genetic counseling, the parents of the fetus chose to terminate the pregnancy.

Case 13: Prenatal ultrasound phenotype was FGR and small kidneys. Karyotype analysis shows 46,XY,del(4)(p15). SNP-array shows arr[hg19] 4p16.3p15.1(68,345-35,252,743)x1. AfymetrixCytoScan 750K Array showed that the severed arm of chromosome 4 contained 105 MM genes and the fragment size was about 35. a gene copy number deletion in p16.3p15.1 of chromosome 4, with a copy number of 1. The involved fragment size was about 35 Mb, containing 105 OMIM genes. Literature and databases have confirmed that this deletion can lead to WolfHirchhom syndrome, with the main clinical manifestations of intrauterine growth restriction, postnatal growth retardation, mental retardation and characteristic facial deformities. Such as armor-like face, more combined with heart and kidney malformations and other congenital abnormalities. The clinical significance is pathogenic. After genetic counseling, the parents of the fetus chose to terminate the pregnancy.

Case 14: Prenatal ultrasound phenotype was FGR, small mandible, and mandible retraction. Karyotype analysis shows 46,XX,add(12)(q24). SNP-array shows arr[hg19]11q23.2q25(113,998,447-134,937,416)x3, 12q24.33(133,718,370-133,777,562)x1. Affymetrix CytoScan 750K Array analysis revealed that chromosome 11q23.2q25 had an increased copy number of 3 with a fragment size of 20.9Mb containing 245 genes. Literature and database confirmed that the genome copy number increases can cause a variety of congenital anomaly: small jaw, cleft lip and palate deformities, growth restriction, feeding difficulties, low muscle tone, spiritual, mental retardation, and special features. The clinical significance is pathogenic. After genetic counseling, the parents of the fetus chose to terminate the pregnancy.

Detailed supplemental material to cases for SNP-array results of fetal FGR with normal karyotype analysis

Case 1: Prenatal ultrasound phenotype was isolated FGR. SNP-array shows arr[hg19]7q11.23(72,723,370-74,143,240)×1. Affymetrix CytoScan 750K Array showed that there was a genomic copy number deletion in 7q11.23 region of chromosome 7. The copy number was 1, and the fragment involved was about 1.42Mb, containing 24 OMIM genes. Among them, ELN deletion mutation is associated with autosomal dominant phenotype of upper dwarf aortic dance stenosis and skin laxa, ELN gene has haploidy underdose effect, and this gene deletion is associated with abnormal phenotype of vascular and connective tissue in 7q11.23 deletion syndrome. This variant covers the region of Williams-Beuren syndrome, which is characterized by mental retardation, short stature, flattening of facial bones, deformities of small teeth, lower lip eversion, aortic valve stenosis, etc. Therefore, the genomic copy number variation in this case is a pathogenic variation. After genetic counseling, the parents of the fetus chose to terminate the pregnancy.

Case 2: Prenatal ultrasound phenotype was FGR and polyhydramnios. SNP-array shows arr[hg19]15q14q21.3(35,077,111-54,347,324)hmz. This case was confirmed by family analysis. Chromosome 15 is maternal paracydiploid [UPD (15) mat], which can cause Prader-WI syndrome, characterized by reduced fetal movement and restricted growth in utero. Infants are mainly manifested as low muscle tone, feeding difficulties and growth retardation, and childhood overappetite, resulting in chronic hypersomnia and obesity, and part of the development of type 2 diabetes. Symptoms typically include mild to moderate mental retardation and learning difficulties, irritability, persistence, and obsessive-compulsive disorder. Forehead stenosis, almond eyes and triangular mouth and other special features, short stature and short hands and feet, male and female all have reproductive organ dysplasia, most are not fertile, some patients have light pigment skin and hair. Maternal parthenodiploid on chromosome 15 is a definite pathogenic variation. After genetic counseling, the parents of the fetus chose to terminate the pregnancy.

Case 3: Prenatal ultrasound phenotype was FGR and echogenic bowel. SNP-array shows arr[hg19]10q11.22q11.23(46,252,072-51,903,756)×1. This case was confirmed by family verification. The increase of fetal chromosome 10 fragment is *de novo*, and there are many reports related to the increase of 10q11.22q11.23 region copy number in literature and database, which may be related to mental retardation, slow growth, heart malformation or nervous system malformation in patients, so it is clinically significant and pathogenic. After genetic counseling, the parents of the fetus chose to terminate the pregnancy.

Case 4: Prenatal ultrasound phenotype was FGR, single umbilical artery, and oligohydramnios. SNP-array shows arr[hg19]8q11.23q12.1(54,456,444-59,599,862)x1. Affymetrix CytoScan 750K Array gene microarray analysis showed that chromosome 8 q11.23 q12.1 had a genome copy number deletion with a size of about 5.1MB. ATP6V1H, RGS20, TCEA1, LYPLA1, MRPL15, SOX17, RP1, XKR4, SBF1P1, LOC105375843, TMEM68, TGS1, LYN, RPS20, SNORD54, MOS, PLAG1, CHCHD7, SDR16C5 and other 36 genes. Searching the database and literature, there were no reports that the deletion of copy number was benign variation, but there were reports on the pathogenicity of similar fragment size deletion, which was mainly manifested as intrauterine growth restriction, slow growth after birth and developmental retardation. The clinical significance of the deletion of copy number in this genome was likely pathogenic. After genetic counseling, the parents of the fetus chose to terminate the pregnancy.

Case 5: Prenatal ultrasound phenotype was FGR, echogenic bowel, mild tricuspid regurgitation, and reverse ductus alpha wave, single umbilical artery, and oligohydramnios. SNP-array shows arr[hg19]6p25.3q27(203,877-170,896,644) x2 hmz. Affymetrix CytoScan 750K Array showed there was heterozygotic deletion on chromosome 6 of the fetus with a copy number of 2, involving the whole chromosome 6. In the pedigree analysis, paternal paraphylodiploid chromosome 6 [UPD (6) pat] and overexpression of PLAG1 and HYMAI imprinted in the 6q24 region are associated with transient neonatal diabetes, which can include intrauterine growth delay, transient neonatal diabetes, macrotongue, and umbilical hernia. Therefore, the clinical significance of this chromosome abnormality is pathogenic. After genetic counseling, the parents of the fetus chose to terminate the pregnancy.

Case 6: Prenatal ultrasound phenotype was FGR and pulmonary stenosis. SNP-array shows arr[hg19]4p16.3p16.1(68,345-6,608,624)×1. Afiymetrix CytoScan 750K showed the short arm p16.3 P16.1 region of chromosome 4 contained 96 genes with a copy number of 1, and the size of the missing fragment was about 6.5Mb. According to literatures and databases, several reports have confirmed that this missing region contains the key pathogenic region of Wolf-Hirschhom syndrome, which can lead to wolf-Hirschhom syndrome, and its main performance is intrauterine growth restriction, postnatal growth retardation, intellectual and mental retardation, characteristic facial deformity, such as armor-like face, often combined with heart and kidney congenital abnormalities such as visceral malformation. The clinical significance of this chromosome abnormality is pathogenic. After genetic counseling, the parents of the fetus chose to terminate the pregnancy.

Case 7: Prenatal ultrasound phenotype was FGR, VSD, and pulmonary valve stenosis with insufficiency. SNP-array shows arr[hg19]15q24.1q24.2(72,965,465-75,567,135)×1. Afiymetrix CytoScan 750K showed there was a copy number deletion on chromosome 15 q24.1q24.2 with a copy number of 1, involving a fragment size of about 2.6Mb and containing 30 OMIM genes. According to the literature review and database, it was confirmed that this section overlaps with 15q24 microdeletion syndrome, and several similar cases were reported to cause a variety of congenital abnormalities, including intrauterine growth delay, short stature, mental retardation, microcephaly, heart malformation, and characteristic facial abnormalities. The clinical significance of this chromosome abnormality is pathogenic. After genetic counseling, the parents of the fetus chose to terminate the pregnancy.

Case 8: Prenatal ultrasound phenotype was FGR, VSD, aortarctia, and left kidney dysplasia. SNP-array shows arr[hg19]16q23.3q24.3(79,800,878-90,146,366)×hmz,16p13.3p12.3(94,807-19,302,326)×hmz. Pedigree analysis of this case confirmed that chromosome 16 was maternal monodiploid [UPD (16) mat], which can cause fetal growth retardation, and the main intrauterine manifestations were reduced fetal movement, restricted growth, heart malformation, and abnormal development of urinary system. Maternal parthenodiploid on chromosome 16 is a definite pathogenic variation. After genetic counseling, the parents of the fetus chose to terminate the pregnancy.

Case 9: Prenatal ultrasound phenotype was FGR, intrahepatic portal shunt, and umbilical vein tumor like dilatation. SNP-array shows arr[hg19]22q11.21(18,648,855-21,459,713)×3. Afiymetrix CytoScan 750K showed that fetal chromosome 22 q11.21 contained 41 OMIM genes, covering 22q11.2 repeat syndrome region, with an increased genome copy number of 2.8MB. By searching the literature and searching the relevant databases, the triple dose score of 22q11.2 in the Clingen database was 3, and the clinical manifestations varied widely from normal phenotypes to a wide range of clinical features. Including growth retardation, intellectual disabilities, learning disabilities, autism, mental disorders, hypatonia, microcephaly, congenital heart disease, hypopharyngeal incomplete closure and other abnormalities. Through pedigree verification, it was confirmed that the copy number of the fetus's genome was duplicated from the mother with normal phenotype, but the 22q11.2 duplication was reported to be pathogenic either from the parent with normal phenotype or from a new mutation, so the clinical significance of the copy number variation was pathogenic. After genetic counseling, the parents of the fetus chose to terminate the pregnancy.

Case 10: Prenatal ultrasound phenotype was FGR, VSD, intrahepatic portal shunt, and umbilical vein tumor like dilatation. SNP-array shows arr[hg19]22q11.21(18,648,855-21,800,471)×1. Afiymetrix CytoScan 750K showed that copy number deletion was found on chromosome 22 q11.21. The deletion size is about 3.1MB, and it contains 87 genes. DGCR6(601279), DGCR2(600594), DGCR14(601755), TBXI1 602054), DGCR8(609030), DGCR6L(609439) lead to the occurrence of 22q11 deficiency syndrome. DiGeorge syndrome can cause the following abnormalities: heart defects, thymus hypoplasia, pharyngopharyngeal and palatal malocclusion with cleft palate, parathyroid dysfunction often with special features of hypocalcaemia, learning and communication difficulties, behavioral, psychological and psychiatric problems in childhood. However, individual patients may not have obvious abnormal clinical manifestations. The clinical significance of the above copy number loss is pathogenic. After genetic counseling, the parents of the fetus chose to terminate the pregnancy.

Case 11: Prenatal ultrasound phenotype was FGR and VSD. SNP-array shows arr[hg19]17p11.2(16,615,982-18,922,171)×3. Afiymetrix CytoScan 750K showed a microduplication on chromosome 17 p11.2, involving a fragment size of about 2.1Mb and containing 21 OMIM genes. The database and literature were checked, and it was found that many reports with similar results were found. The microduplication fragments all contain RAI1 gene, and the corresponding encoding product of RAI1 is Retinoic acid-induced gene protein 1(RIG-I), which can lead to the occurrence of Potocki-Lupski syndrome. The main clinical characteristics of the patients are as follows, mild to moderate mental retardation, mental retardation, short stature, inattention, autism, hyperactivity disorder, triangular face, high zygomatic arch, frontal eminence, palatal dysplasia, abnormal heart development, etc.. So the clinical significance is is pathogenic. After genetic counseling, the parents of the fetus chose to terminate the pregnancy.

Case 12: Prenatal ultrasound phenotype was FGR, persistent left superior vena cava, and renal parenchyma echo enhancement. SNP-array shows arr[hg19]2p25.3p11.2(50,813-87,053,152)hmz,arr[hg19]2q11.1q37.3(95,550,957-242,773,583) hmz. Afiymetrix CytoScan 750K showed there was heterozygous deletion of the whole chromosome on chromosome 2, and it was confirmed by family analysis that chromosome 2 was maternal and parparent diploid. By searching the literature and database, it was speculated that there might be imprinted genes on chromosome 2 staining, and its pathogenicity was unknown. Some reports suggest that chromosome 2 maternal parthenodiploid may be associated with fetal growth restriction, growth retardation, hypospadias and oligohydramnios. Parthenodiploids such as chromosome 2 increase the risk of homozygous disease caused by recessive pathogenic genes on maternal chromosome 2. After genetic counseling, the parents of the fetus chose to terminate the pregnancy.

Case 13: Prenatal ultrasound phenotype was isolated FGR. SNP-array shows arr[hg19]22q11.21(18,648,855-21,800,471)×3. Afiymetrix CytoScan 750K showed that fetal chromosome 22 q11.21 contained 43 OMIM genes, covering 22q11.2 microduplication syndrome region, with an increased genome copy number of 3.1MB. By searching the literature and searching the relevant databases, the triple dose score of 22q11.2 in the Clingen database was 3, and the clinical manifestations varied widely from normal phenotypes to a wide range of clinical features. Including growth retardation, intellectual disabilities, learning disabilities, autism, mental disorders, hypatonia, microcephaly, congenital heart disease, hypopharyngeal incomplete closure and other abnormalities. Through pedigree verification, it was confirmed that the copy number of the fetus's genome was duplicated from the father with normal phenotype. After genetic counseling, the parents of the fetus chose to term delivery.

Case 14: Prenatal ultrasound phenotype was isolated FGR. SNP-array shows arr[hg19]4q28.3q31.3(133,718,289-154,569,367)hmz. Afiymetrix CytoScan 750K showed that there was heterozygous deletion in q28.3q31.3 region of chromosome 4, involving a fragment length of about 20.8Mb, containing 49 OMIM genes. Among them, RAB33B(605950) and MAB21L2(604357) are recessive pathogenic genes. Homozygous or double heterozygous mutations can lead to the occurrence of short limb deformity in patients, and the loss of heterozygous in this case increases the risk of disease of the two genes. The parents refused to do pedigree testing. After genetic counseling, the parents of the fetus chose to term delivery.

Case 15: Prenatal ultrasound phenotype was isolated FGR. SNP-array shows arr[hg19]15q24.1q24.2(72,969,435-75,601,120)x3. Afiymetrix CytoScan 750K showed duplication of genomic copy number in q24.1q24.2 region of fetal chromosome 15, with a copy number of 3, involving a fragment size of about 2.6Mb, including BBS4 (600374), HCN4 (605206), NPTN (612820), NPTN-IT1 (615176),LOXL1-AS1 (616800), PML (102578), CYP11A1 (118485), SEMA7A (607961) and 32 OMIM genes. According to literature review and database, this repeated fragment almost completely overlaps with 15q24 recurrent region (A-C). ClinGen database evaluated that the triple dose sensitivity score of 15q24 region (A-C) was 1 point. At present, there are few reports of repeated cases in this region with variable clinical phenotypes, including developmental delay, high muscle tone, and craniofacial abnormalities. Cardiac abnormalities and other malformations (PMIDs: 19557438, 20860070). ClinVar database showed a similar case report with a clinical presentation of overall developmental delay. No benign reports like this repetition were found in DGV database. The results of pedigree validation showed that the fetal genome copy number was duplicated and inherited from the normal phenotype of the mother. In summary, the clinical significance of duplicate genome copy number is VUS. After genetic counseling, the parents of the fetus chose to term delivery.

Case 16: Prenatal ultrasound phenotype was isolated FGR. SNP-array shows arr[hg19]14q12(25,364,014-26,860,808)x3. Afiymetrix CytoScan 750K showed that there was an increased copy number of 3 in the q12 region of fetal chromosome 14, involving a fragment size of about 1.4Mb and containing one OMIM gene: STXBP6 (607958). No increase of copy number in related regions was reported in literature and database. At present, it is not clear whether the increase of copy number is pathogenic, so its clinical significance is VUS. After genetic counseling, the parents of the fetus chose to term delivery.

Case 17: Prenatal ultrasound phenotype was FGR and VSD. SNP-array shows arr[hg19]4q24(106,284,925-107,545,257)×3. Afiymetrix CytoScan 750K showed there was an increase in the number of vickers on chromosome 6 q24, and the number of shell carriers was 3. The involved fragment size was about 1.2Mb, containing 4 OMIM genes, respectively; PPA2, INTSI2, NPNT, AIMP1. A search of databases and literature showed that AIMPI basal country mutation could induce leukodystrophy, but there were no reports of an increase in the number of patients. Its clinical significance is VUS. This case was lost to follow-up.

Case 18: Prenatal ultrasound phenotype was isolated FGR. SNP-array shows arr[hg19]18p11.23p11.22(7,153,845-8,964,650)x3. Afiymetrix CytoScan 750K showed duplication of genome copy number in p11.23 p11.22 region of fetal chromosome 18, with copy number of 3, involving fragment size of about 1.8Mb, including PTPRM (176888), RAB12 (616448), GACAT2 (616131), MTCL1 (615766) OMIM genes. ClinGen database did not show that this region contained triple dose sensitive genes. No benign reports like this repetition were found in DGV database. No case report similar to this variant fragment was found in DECIPHER and ClinVar databases. The results of pedigree validation showed that fetal genome copy numbers were repeatedly inherited from fathers with normal phenotypes. In summary, the duplicated genome copy number variants were classified as likely benign. After genetic counseling, the parents of the fetus chose to term delivery.

Case 19: Prenatal ultrasound phenotype was FGR and tricuspid regurgitation. SNP-array arr[hg19]4q35.2(188,155,530-190,957,460)x1. Afiymetrix CytoScan 750K showed that there was a deletion of genomic copy number in q35.2 region of fetal chromosome 4 with a copy number of 1, involving a fragment size of about 2.8Mb and containing 3 OMIM genes. After checking the literature and database, there was no relevant report about whether the missing part was pathogenic or not. This case has been verified by family verification: chromosome 4 with fetal deletion mutation was inherited from the mother with normal phenotype. So the clinical significance wae classified as likely benign. After genetic counseling, the parents of the fetus chose to term delivery.
